# For the love of all that is holy, stop prescribing sodium valproate and carbamazepine together

**DOI:** 10.12669/pjms.39.1.7281

**Published:** 2023

**Authors:** Wajid Jawaid

**Keywords:** Sodium valproate, Carbamazepine, Anti-seizure drugs, Hepatic enzyme inducers, Hepatic enzyme inhibitors

## Abstract

Sodium valproate and carbamazepine are two time-tested drugs for treatment of epilepsy. Individually, they are usually excellent choices in treating a broad spectrum of epileptic seizures. They are, however, not friendly with each other. Their co-administration affects the drug levels of each other by influencing the action of hepatic enzymes. This write up attempts to give an overview of the mechanism of this drug interaction, and informs readers why this combination is a bad choice. The author hopes that this will help in raising awareness among the physicians regarding the dangers of this prescription, and in putting a full stop to this practice.

A young epileptic patient comes to a general physician. He complains that his fits are not fully controlled despite taking his prescribed 500 mg sodium valproate tablet twice daily. The physician thinks there is an easy solution to the problem, and nonchalantly adds 200 mg carbamazepine tablet twice daily. Next patient please.

This is a very common scenario that reflects a lack of awareness among the physicians as regards the treatment of epilepsy. The above-mentioned scenario actually demonstrates two errors in the young man’s latest prescription. First is unnecessary combination therapy, without making an utmost effort to keep the patient on a single anti-seizure drug. This discussion is for some other day though. The aim of this piece is to draw the readers’ attention towards another problematic approach. You know where I am going with this, you have read the title.

The fact that sodium valproate and carbamazepine combination is a bad choice is known at least since 1982, before many of the readers were even born.^1^ Though the chemical reactions in the body are usually not as straightforward, a simple understanding of the effect of these drugs on hepatic enzymes will explain why this combination is a recipe for failure.^2^ Sodium valproate, as an inhibitor of hepatic enzymes, interferes in carbamazepine metabolism; this often results in increased serum levels of carbamazepine.^3^ Carbamazepine, on the other hand, is an inducer of hepatic enzymes; co-administration of carbamazepine with sodium valproate therefore tends to decrease the serum levels of the latter.^4^ This combination is likely to ensure that carbamazepine reaches toxic serum levels and sodium valproate remains at a sub-therapeutic level in serum. If the combination is necessary for some unavoidable reason (like some drug-resistant epilepsies), the serum levels of both these drugs must be closely monitored.^5^ This, however, is an exception. From a general standpoint, this combination is good only if you want to teach someone the meaning of a *lose-lose situation*.

Sodium valproate-carbamazepine combination is anything but responsible. When it comes to a life threatening condition like seizures, it becomes outright dangerous. Playing with dangers may be mantra of a killer, not a supposed messiah.
